# Supplementary material for: Methylation of all BRCA1 copies predicts response to the PARP inhibitor rucaparib in ovarian carcinoma
Source: Nat Commun. 2018 Sep 28;9:3970. doi: 10.1038/s41467-018-05564-z (PMC6162272; doi:10.1038/s41467-018-05564-z)
Supplement: Supplementary file 1 — Supplementary Information [file 41467_2018_5564_MOESM1_ESM.pdf]

Supplementary Information

Methylation of all *BRCA1* copies predicts response to the PARP inhibitor rucaparib in ovarian carcinoma

Kondrashova *et al.*

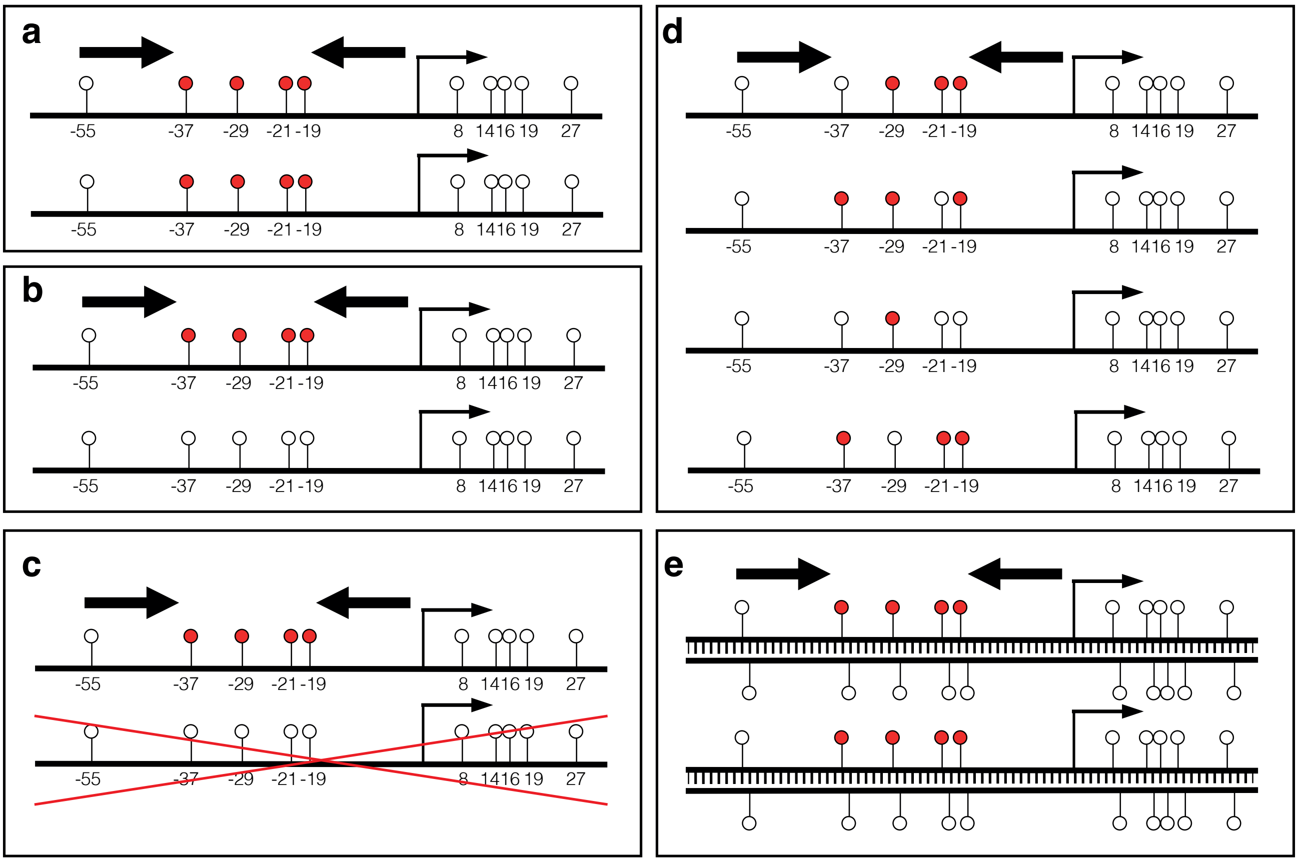
a
-55 -37 -29 -21 -19 8 1416 19 27
-55 -37 -29 -21 -19 8 1416 19 27
b
-55 -37 -29 -21 -19 8 1416 19 27
-55 -37 -29 -21 -19 8 1416 19 27
c
-55 -37 -29 -21 -19 8 1416 19 27
-55 -37 -29 -21 -19 8 1416 19 27
d
-55 -37 -29 -21 -19 8 1416 19 27
-55 -37 -29 -21 -19 8 1416 19 27
-55 -37 -29 -21 -19 8 1416 19 27
-55 -37 -29 -21 -19 8 1416 19 27
e

Supplementary Figure 1. Potential methylation states at the *BRCA1* locus. a Homozygous methylation, where any number of copies of *BRCA1* are present and all copies are methylated. **b** Heterozygous methylation, where two or more copies of *BRCA1* are present and at least one copy is unmethylated. **c** Homozygous null (nullizygous), where only one copy is methylated due to loss of the other copy (including large scale aneuploidy events), also known as hemizygous methylation. **d** Partial methylation describes an inconsistent methylation state amongst multiple CpGs in the same copy, with both methylated CpGs and unmethylated CpGs being present. **e** Hemi-methylated is a term that is used in some publications to describe heterozygous methylation, but is more frequently used in the epigenetic literature to describe methylation of one of two strands of DNA during replication or transcription. In some systems hemi-methylation has been shown to be transcriptionally activating [1]. Hemi-methylated should not be confused with hemizygous methylation, which is alternative description of a compound heterozygote of a methylated and a deletion copy.

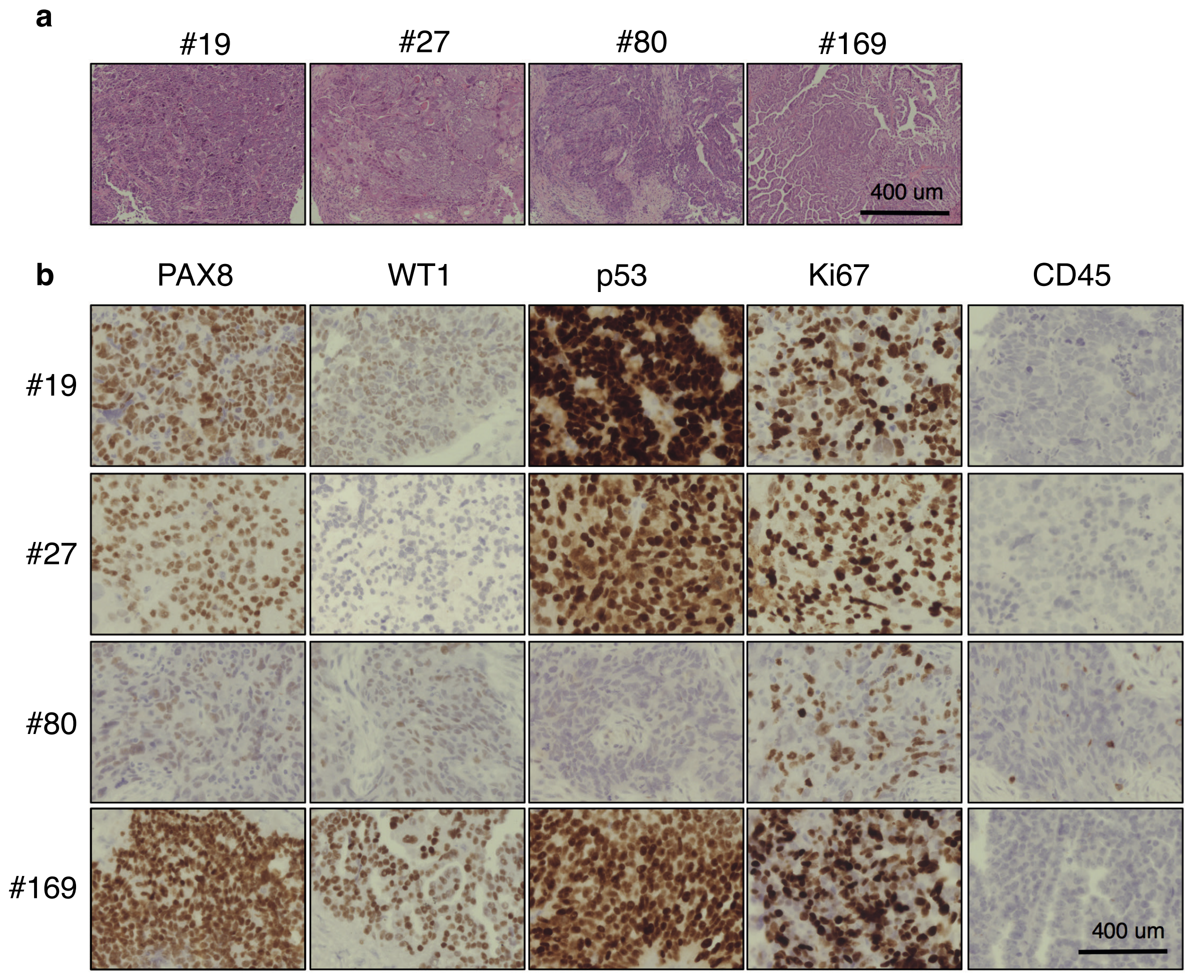
a
#19
#27
#80
#169
400 um
b
PAX8
WT1
p53
Ki67
CD45
#19
#27
#80
#169
400 um

Supplementary Figure 2. Histopathologic characteristics of four HGSOC PDX. Passage 1 (T1) PDX tumor samples were H&E stained (**a)** and were stained by IHC for p53, WT1 and PAX8 (**b)**. Protein expression was similar for matched baseline HGSOC and PDX samples.

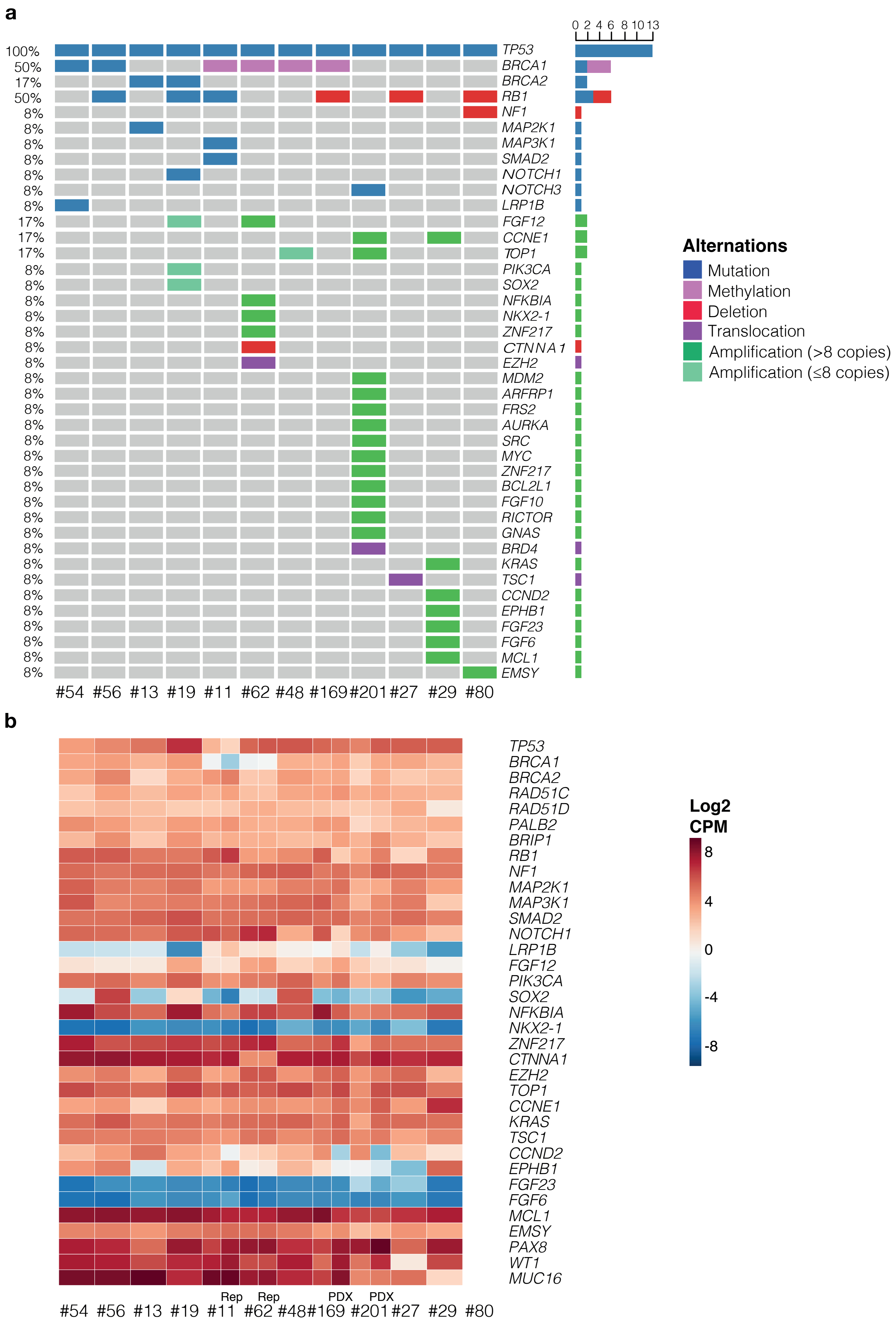
a
0 2 4 6 8 10 13
100% TP53
50% BRCA1
17% BRCA2
50% RB1
8% NF1
8% MAP2K1
8% MAP3K1
8% SMAD2
8% NOTCH1
8% NOTCH3
8% LRP1B
17% FGF12
17% CCNE1
17% TOP1
8% PIK3CA
8% SOX2
8% NFKBIA
8% NKX2-1
8% ZNF217
8% CTNNA1
8% EZH2
8% MDM2
8% ARFRP1
8% FRS2
8% AURKA
8% SRC
8% MYC
8% ZNF217
8% BCL2L1
8% FGF10
8% RICTOR
8% GNAS
8% BRD4
8% KRAS
8% TSC1
8% CCND2
8% EPHB1
8% FGF23
8% FGF6
8% MCL1
8% EMSY
#54 #56 #13 #19 #11 #62 #48 #169 #201 #27 #29 #80
Alternations
Mutation
Methylation
Deletion
Translocation
Amplification (>8 copies)
Amplification (≤8 copies)
b
TP53
BRCA1
BRCA2
RAD51C
RAD51D
PALB2
BRIP1
RB1
NF1
MAP2K1
MAP3K1
SMAD2
NOTCH1
LRP1B
FGF12
PIK3CA
SOX2
NFKBIA
NKX2-1
ZNF217
CTNNA1
EZH2
TOP1
CCNE1
KRAS
TSC1
CCND2
EPHB1
FGF23
FGF6
MCL1
EMSY
PAX8
WT1
MUC16
Log2 CPM
8
4
0
-4
-8
Rep Rep PDX PDX
#54 #56 #13 #19 #11 #62 #48 #169 #201 #27 #29 #80

Supplementary Figure 3. Genomic profiling of 12 HGSOC PDX. **a** Genomic events detected by the Foundation Medicine T5a test, BROCA assay and *BRCA1* promoter methylation testing. The Foundation Medicine T5a test was performed on PDX samples, except for case #48, where it was performed on baseline material. T5a test results and BROCA v4 assay results for PDX #11, #13, #27, #29, #56, #62 were previously published; BROCA v6 was performed for all other PDX [2]. **b** RNA-seq gene expression for genes with detected mutations or copy number changes. RNA-seq was performed on baseline patient HGSOC material samples. RNA-seq was also performed on PDX #169 and #201 samples, to verify expression levels observed in the matched HGSOC with suboptimal sample quality due to either low neoplastic cellularity or poor RNA quality (#80 inadequate quality).

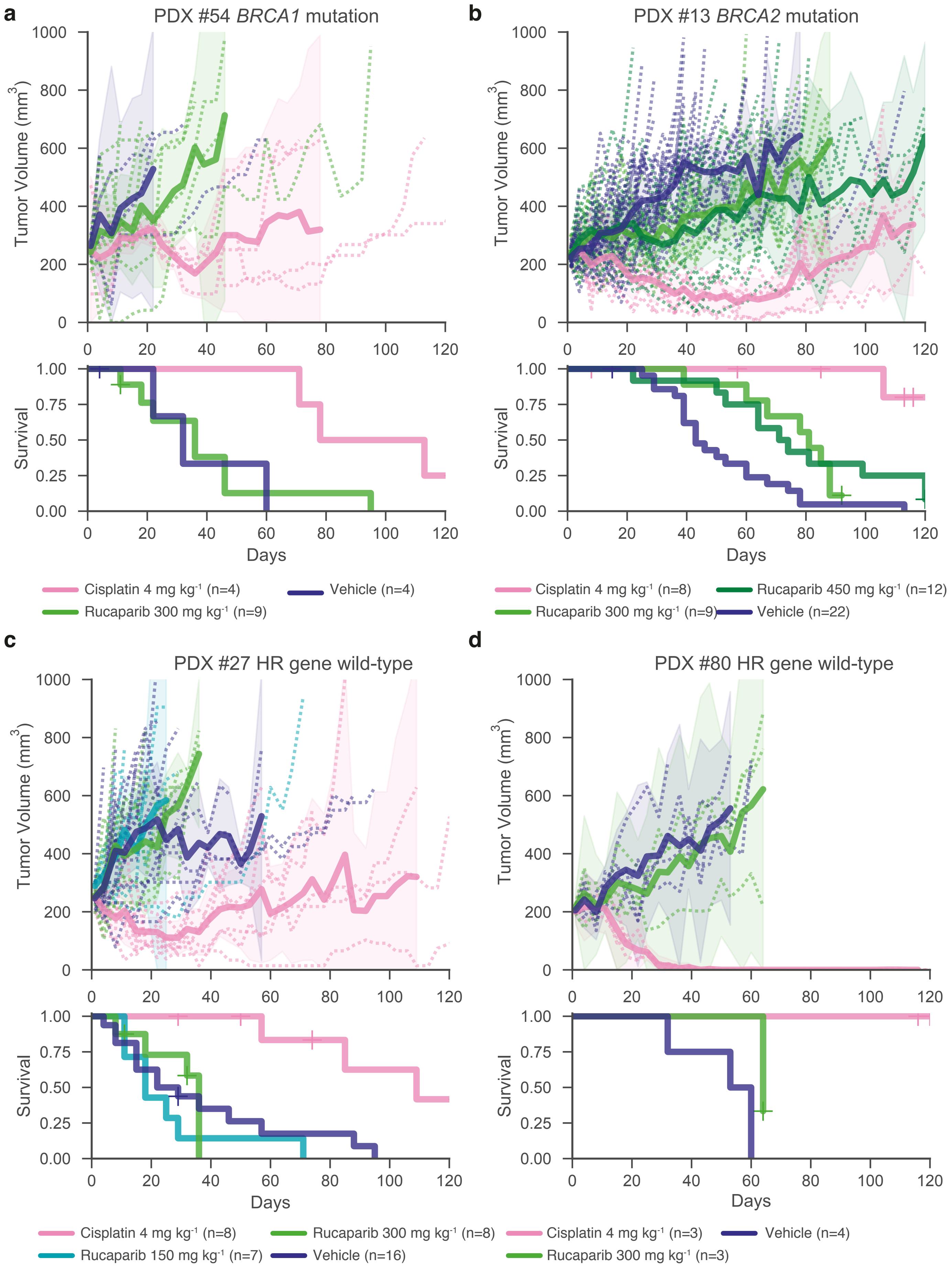
a
PDX #54 BRCA1 mutation
Tumor Volume (mm3)
Survival
Days
Cisplatin 4 mg kg-1 (n=4)
Vehicle (n=4)
Rucaparib 300 mg kg-1 (n=9)
b
PDX #13 BRCA2 mutation
Tumor Volume (mm3)
Survival
Days
Cisplatin 4 mg kg-1 (n=8)
Rucaparib 450 mg kg-1 (n=12)
Rucaparib 300 mg kg-1 (n=9)
Vehicle (n=22)
c
PDX #27 HR gene wild-type
Tumor Volume (mm3)
Survival
Days
Cisplatin 4 mg kg-1 (n=8)
Rucaparib 300 mg kg-1 (n=8)
Rucaparib 150 mg kg-1 (n=7)
Vehicle (n=16)
d
PDX #80 HR gene wild-type
Tumor Volume (mm3)
Survival
Days
Cisplatin 4 mg kg-1 (n=3)
Vehicle (n=4)
Rucaparib 300 mg kg-1 (n=3)

Supplementary Figure 4. *In vivo* response to cisplatin and rucaparib treatment in additional *BRCA1/2* mutant versus HR-DNA repair gene wild-type PDX. Rucaparib and cisplatin response in **a** *BRCA2* mutant PDX #54; **b** *BRCA1* mutant PDX #13; **c-d** HR-DNA repair gene wild-type PDX #27 and PDX #80. Recipient mice bearing PDX were randomized to treatment with vehicle or rucaparib, at the dose shown. PDX were harvested at tumor volume of 600-700 mm^3. Cisplatin response data for PDX #54 and #27 were previously published [2]. See Table 1 and Supplementary Table 2 for median TTH and p-values for survival comparison. Mean tumor volume (mm^3) ±95% CI and corresponding Kaplan-Meier survival analysis. Censored events are represented by crosses on Kaplan-Meier plot. n= individual mice. Of note, the chemo-naïve PDX #80 harbored focal amplification of *EMSY*, a proposed regulator of HR pathway, but was refractory to rucaparib. Association of HR deficiency with *EMSY* amplification has been controversial, with some reports of *EMSY* amplification downregulating HR activity, possibly through downregulation of *BRCA2* [3, 4]. In keeping with our data, another report suggests that *EMSY* amplification does not affect the formation of RAD51 foci and, furthermore, does not sensitize the cells to PARP inhibitors [5].

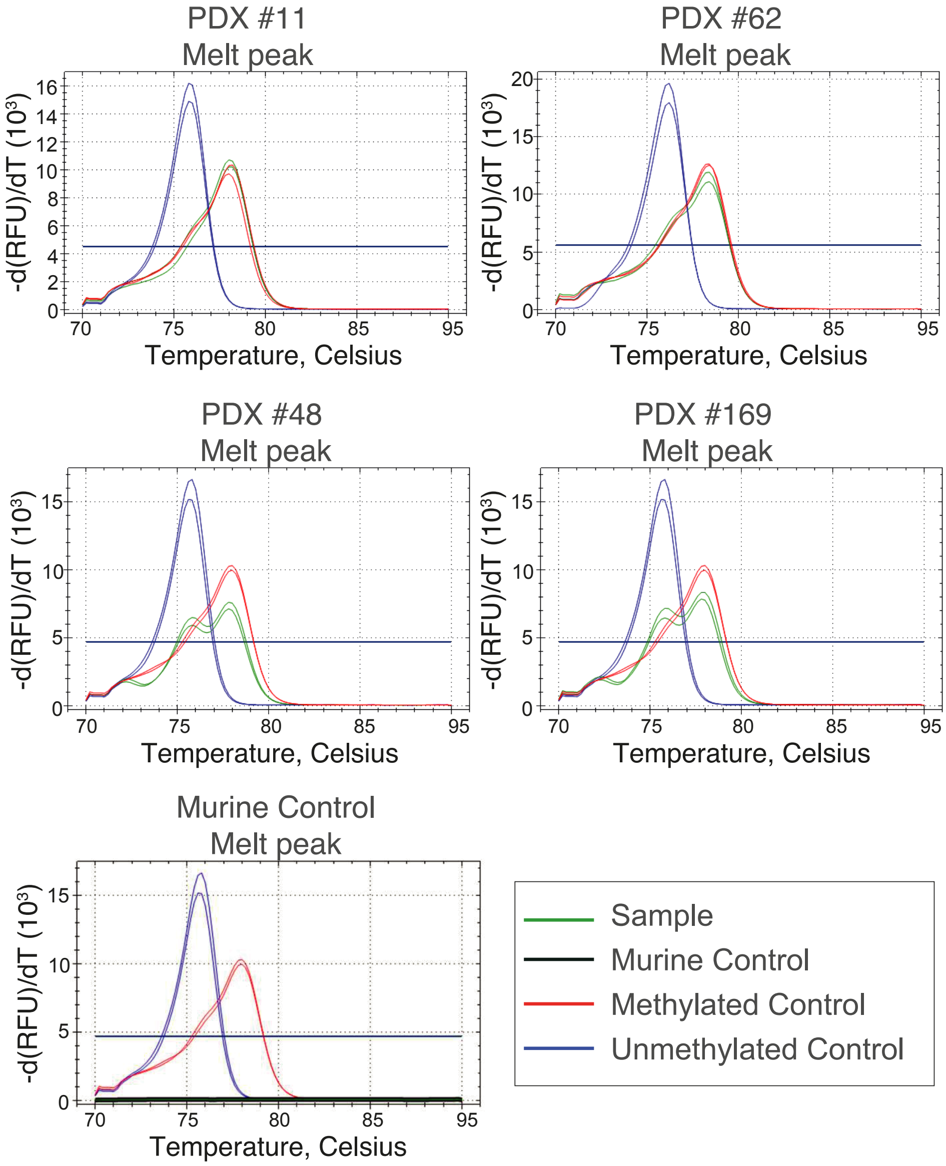
PDX #11
Melt peak
-d(RFU)/dT (10³)
Temperature, Celsius
PDX #62
Melt peak
-d(RFU)/dT (10³)
Temperature, Celsius
PDX #48
Melt peak
-d(RFU)/dT (10³)
Temperature, Celsius
PDX #169
Melt peak
-d(RFU)/dT (10³)
Temperature, Celsius
Murine Control
Melt peak
-d(RFU)/dT (10³)
Temperature, Celsius
Sample
Murine Control
Methylated Control
Unmethylated Control

Supplementary Figure 5. Analysis of *BRCA1* methylation by MS-HRM assay in four PDX models with *BRCA1* methylation. Methylation specific high resolution melt analysis of four CpG sites in the *BRCA1* promoter. In fully methylated control DNA (red) 5-Methylcytosines are not bisuphite converted to uracil, resulting in a higher melting temperature PCR product than in the unmethylated control (blue) in which all cytosines are converted to uracil. Samples from PDX #11 and #62 show melt curves concordant with the methylated control, indicating full methylation at the four sites assayed in all copies of *BRCA1* (Homozygous methylation). PDX #48 and PDX #169 have melt curves with two distinct peaks corresponding to the methylated and unmethylated controls, indicating a mixture of fully methylated and unmethylated copies in approximately equal abundance (Heterozygous methylation). The murine control shows no melt peak confirming that mouse material does not contribute any signal in the xenograft samples.

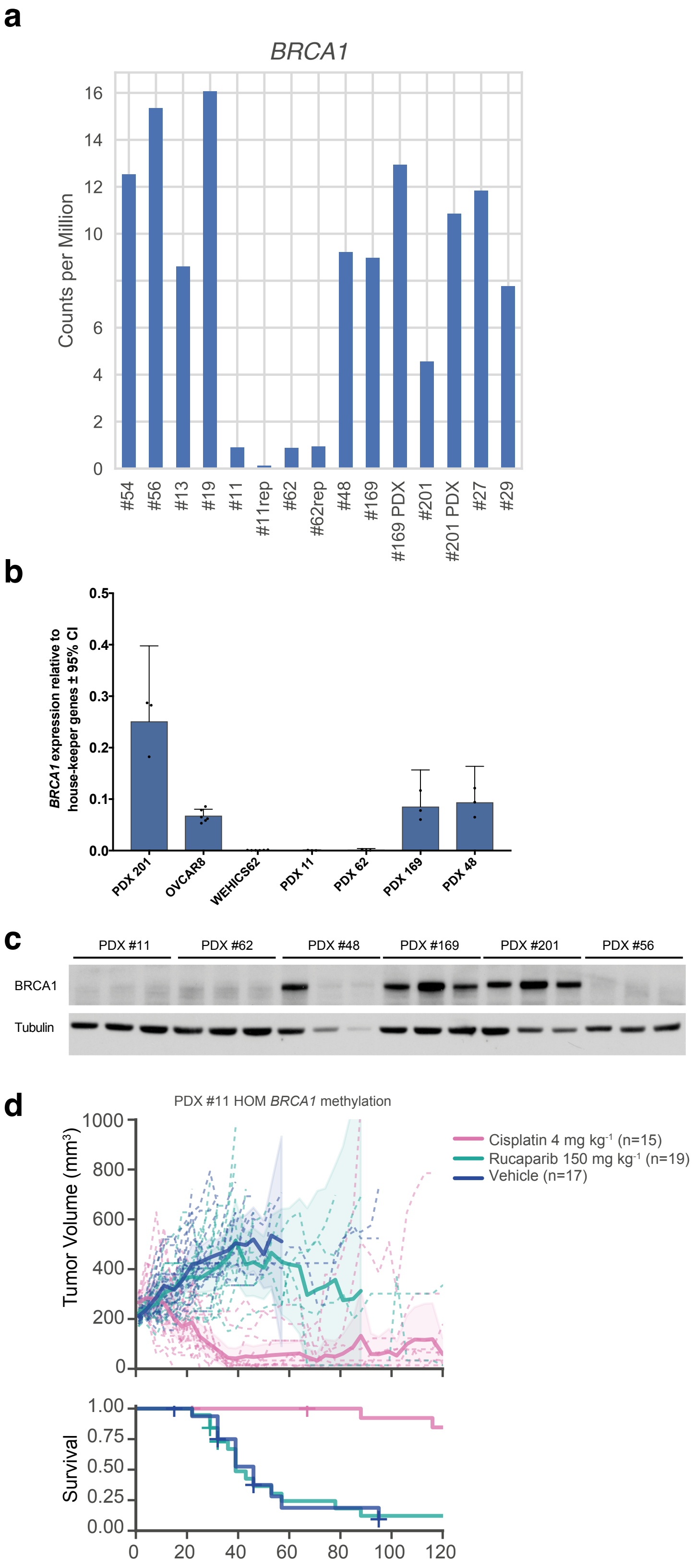
a
BRCA1
Counts per Million
0
2
4
6
10
12
14
16
#54
#56
#13
#19
#11
#11rep
#62
#62rep
#48
#169
#169 PDX
#201
#201 PDX
#27
#29
b
BRCA1 expression relative to house-keeper genes ± 95% CI
0.0
0.1
0.2
0.3
0.4
0.5
PDX 201
OVCAR8
WEHICS62
PDX 11
PDX 62
PDX 169
PDX 48
c
PDX #11
PDX #62
PDX #48
PDX #169
PDX #201
PDX #56
BRCA1
Tubulin
d
PDX #11 HOM BRCA1 methylation
Tumor Volume (mm³)
0
200
400
600
800
1000
Cisplatin 4 mg kg⁻¹ (n=15)
Rucaparib 150 mg kg⁻¹ (n=19)
Vehicle (n=17)
Survival
0.00
0.25
0.50
0.75
1.00
0
20
40
60
80
100
120

Supplementary Figure 6. Silencing of *BRCA1* by methylation of *BRCA1* promoter in four *BRCA1*-methylated HGSOC. a *BRCA1* expression assessed by RNA-seq in four HGSOC that gave rise to PDX (#11, #62, #48, #169). HGSOC samples with homozygous *BRCA1* methylation (#11 and #62) had reduced *BRCA1* expression while samples with heterozygous *BRCA1* methylation did not (#48 and #169). Since baseline ascites sample #169 low tumor purity of about 15% (estimated by *TP53* sequencing of the matched DNA sample), a PDX sample was also sequenced (T3). **b** *BRCA1* expression assessed by qRT-PCR in four *BRCA1* methylated PDX models: #11, #62, #48, #169 and two cell line models: WEHICS62 and OVCAR8. *BRCA1* expression is assessed relative to mean expression of four house-keeper genes. **c** BRCA1 protein expression assessed by Western Blotting in four *BRCA1* methylated PDX models: #11, #62, #48, #169; a HR-competent PDX model #201; and a *BRCA1* mutated PDX model #56. We were unable to accurately assess BRCA1 protein expression in replicates 2 and 3 of PDX model #48 by WB due to low lysate concentrations (Supplementary Table 5). **d** Response to cisplatin and rucaparib 150 mg kg^{-1} of PDX #11 with homozygous *BRCA1* methylation *in vivo* (higher doses were not tested).

a

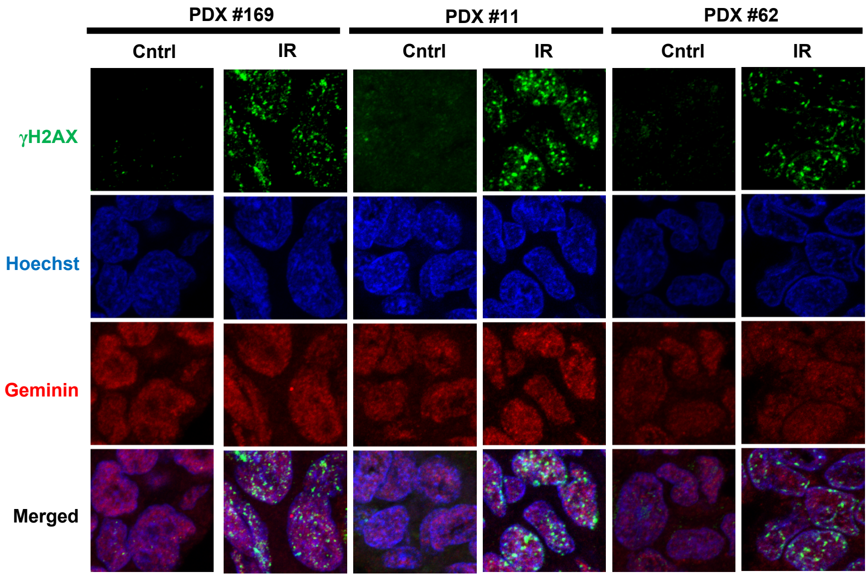
PDX #169
PDX #11
PDX #62
Cntrl
IR
Cntrl
IR
Cntrl
IR
γH2AX
Hoechst
Geminin
Merged

b

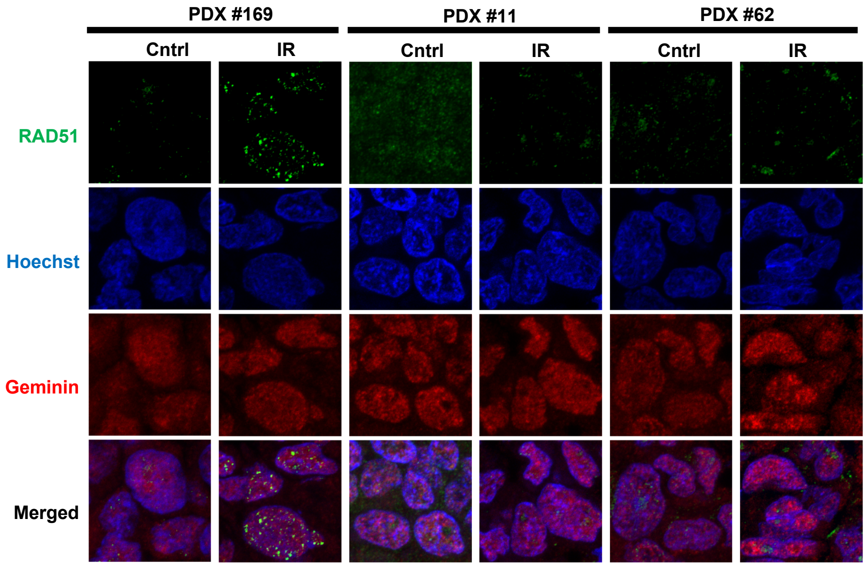
PDX #169
PDX #11
PDX #62
Cntrl
IR
Cntrl
IR
Cntrl
IR
RAD51
Hoechst
Geminin
Merged

Supplementary Figure 7. Ex vivo γH2AX (a) and RAD51 (b) foci formation in three PDX models with *BRCA1* methylation. Panel **b** is also shown in Fig. 3c, and is repeated in this figure for ease of interpretation. γH2AX foci are observed at the sites of DNA damage, and RAD51 foci are observed at the sites of HR pathway repair.

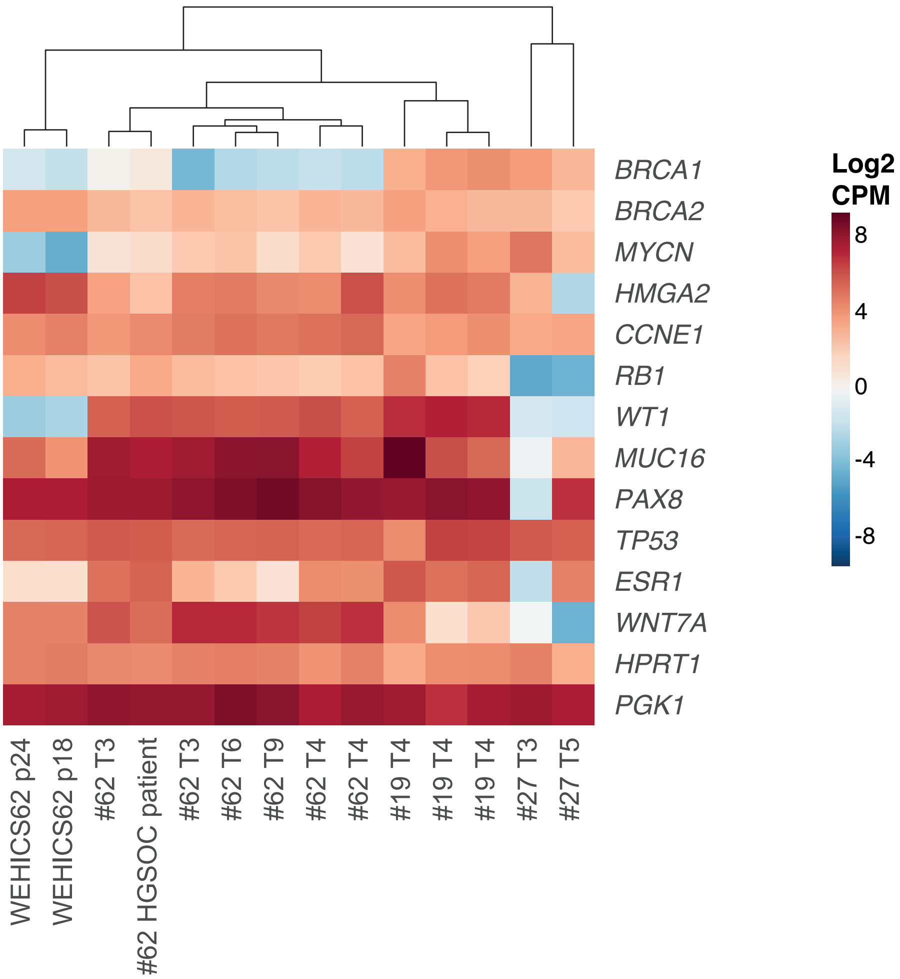
BRCA1
BRCA2
MYCN
HMGA2
CCNE1
RB1
WT1
MUC16
PAX8
TP53
ESR1
WNT7A
HPRT1
PGK1
WEHICS62 p24
WEHICS62 p18
#62 T3
#62 HGSOC patient
#62 T3
#62 T6
#62 T9
#62 T4
#62 T4
#19 T4
#19 T4
#19 T4
#27 T3
#27 T5
Log2 CPM
8
4
0
-4
-8

Supplementary Figure 8. RNA-seq analysis of the WEHICS62 cell line and matched PDX #62 T3-T8 samples in comparison with non-*BRCA1* methylated PDX samples. *BRCA1* mRNA expression was reduced in samples from case #62 and PDX #62 with homozygous *BRCA1* methylation, in comparison to PDX without *BRCA1* methylation: PDX #19 with a *BRCA2* mutation and PDX #27 which was HR-DNA repair gene wild-type.

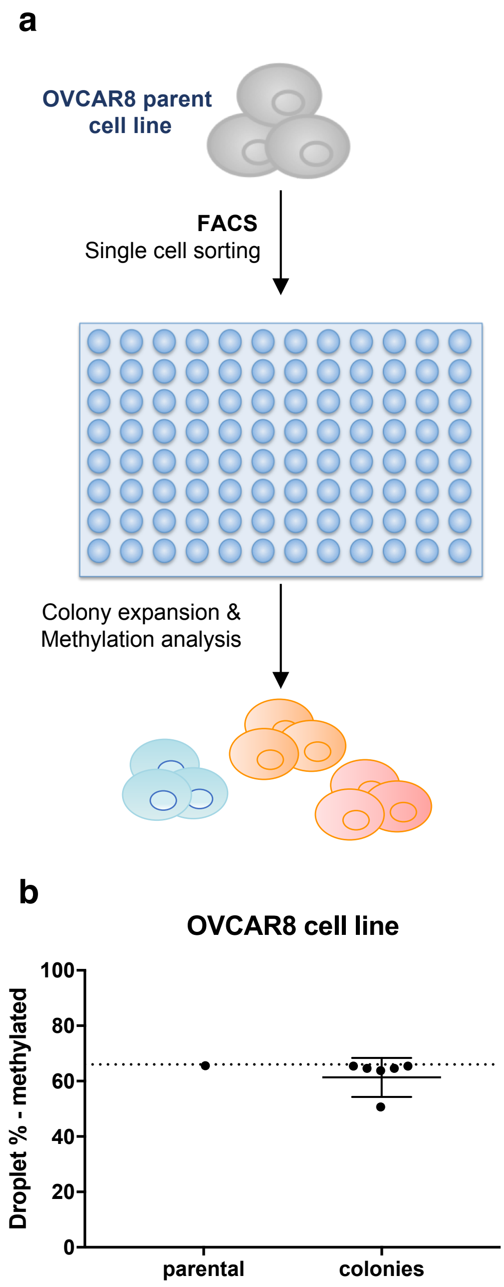
a
OVCAR8 parent
cell line
FACS
Single cell sorting
Colony expansion &
Methylation analysis
b
OVCAR8 cell line
Droplet % - methylated
100
80
60
40
20
0
parental
colonies

Supplementary Figure 9. *BRCA1* methylation analysis in OVCAR8 single-cell colonies. a A schematic showing how OVCAR8 single-cell colonies were obtained. **b** *BRCA1* methylation analysis by MS-ddPCR in OVCAR8 single-cell colonies shows consistently similar percentage of *BRCA1* methylation (Median=64%) as detected in the parental OVCAR8 cell line (65.2%), supporting the hypothesis that OVCAR8 has two methylated copies of *BRCA1* and one unmethylated.

a

b

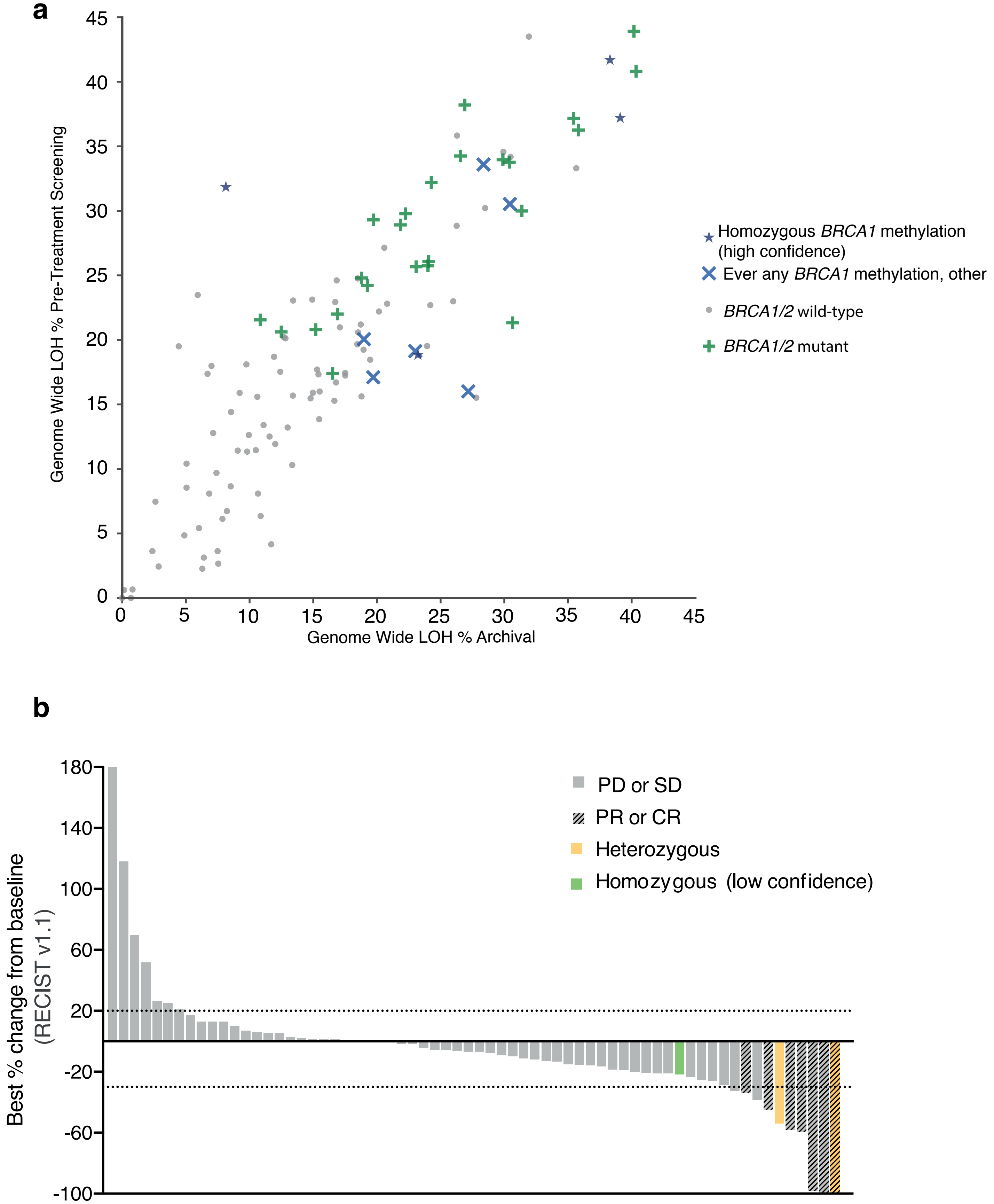

Genome Wide LOH % Pre-Treatment Screening
Genome Wide LOH % Archival
0
5
10
15
20
25
30
35
40
45
Homozygous BRCA1 methylation (high confidence)
Ever any BRCA1 methylation, other
BRCA1/2 wild-type
BRCA1/2 mutant
Best % change from baseline (RECIST v1.1)
180
140
100
60
20
-20
-60
-100
PD or SD
PR or CR
Heterozygous
Homozygous (low confidence)

Supplementary Figure 10. Association of homozygous *BRCA1* promoter methylation and rucaparib response in the ARIEL2 Part 1 trial. a Concordance of genomic LOH score for pre-treatment and archival samples. Analysis of HGSOC cases with homozygous *BRCA1* methylation in the pre-treatment tumor biopsy, which was of high confidence based on adequate neoplastic cellularity (homozygous *BRCA1* methylation (high confidence)), compared with patients with HGSOC in which there had ever been any other evidence of *BRCA1* methylation (ever any *BRCA1* methylation), compared with all other patients in the ARIEL2 Part 1 trial without any *BRCA1* methylation (*BRCA1/2* mutant versus *BRCA1/2* wild-type non-*BRCA1* methylated subgroups). In most cases genomic LOH scores were highly concordant between archival and pre-treatment biopsy samples; however, for one case an increase in LOH score was observed from the archival to screening sample (8.14% to 31.84%), potentially due to ongoing HR deficiency and accumulation of genomic scarring during this interval. **b** Best percentage change from baseline in sum of longest diameter of target lesions according to RECIST in *BRCA*1/2 wild-type LOH low subgroup of patients by *BRCA1* methylation status. Each bar represents percentage change from baseline in sum of the longest diameter of target lesions for an individual patient according to RECIST. In some patients although best percentage change of > 30% was observed, the response was not investigator confirmed and thus was classified as SD or PD by RECIST1.1 criteria.

Supplementary Table 1. Estimated nuclear extract and protein lysate concentrations for PDX tumor samples analysed by BRCA1 WB. We were unable to accurately assess BRCA1 protein expression in replicates 2 and 3 of PDX model #48 by WB (Supplementary Fig. 6c) due to low lysate concentrations.

PDX model	Replicate	Nuclear Extract (mg ml^{-1})	Lysate (mg ml^{-1})
11	1	0.57	0.71
11	2	0.82	0.71
11	3	1.3	1
62	1	1.55	1
62	2	1.94	1
62	3	2.52	1
48	1	1.6	1
48	2	0.52	0.51
48	3	0.28	0.45
169	1	1.62	1
169	2	3.33	1
169	3	1.95	1
201	1	1.41	1
201	2	0.96	0.8
201	3	0.58	0.65
56	1	0.71	0.77
56	2	1.87	1
56	3	1.82	1

Supplementary Table 2. *BRCA1* methylation assessed by MS-ddPCR in the ovarian cancer cell lines, OVCAR8, OVCAR8 derivative with *RAD51C* KO and WEHICS62.

Sample	*BRCA1* methylation (%)	*BRCA1* methylation zygosity
**OVCAR8**	65.2	heterozygous
**OVCAR8 *RAD51C* KO**	66.1	heterozygous
**WEHICS62**	100	homozygous

BRCA1 promoter methylation was assessed by MS-ddPCR for three cell lines and the zygosity state imputed as either heterozygous or homozygous.

Supplementary Table 3. *BRCA1* copy number in the four *BRCA1* methylated PDX and two cell line models assessed by MLPA-Seq assay.

Model	*BRCA1* methylation zygosity	Mean *BRCA1* exon ratio ± SD	Estimated *BRCA1* copy number
**PDX #11**	Homozygous	0.46 ± 0.06	1
**PDX #62**	Homozygous	0.86 ± 0.09	2
**PDX #48**	Heterozygous	0.83 ± 0.09	2
**PDX #169**	Heterozygous	1.03 ± 0.28	2
**Cell line OVCAR8**	Heterozygous	1.48 ± 0.18	3
**Cell line OVCAR8 *RAD51C* KO**	Heterozygous	1.31 ± 0.17	3
**Cell line WEHICS62**	Homozygous	1.12 ± 0.14	2

Supplementary Table 4. Best confirmed overall response by *BRCA1* promoter methylation status in the ARIEL2 Part 1 trial.

Best overall response	Homozygous *BRCA1* methylation high confidence N (%)	*BRCA1/2* wild-type non-*BRCA1* methylated N (%)	Ever any *BRCA1* methylation other N (%)	*BRCA1/2* mutated non-*BRCA1* methylated N (%)
CR or PR	5 (83%)	24 (17%)	5 (33%)	32 (80%)
SD or PD	1 (17%)	112 (78%)	9 (60%)	6 (15%)
NE or ongoing	-	7 (5%)	1 (7%)	2 (5%)
**Total**	6	143	15	40

Best confirmed overall response to rucaparib (RECIST1.1) for all cases where homozygous *BRCA1* methylation could be confirmed in the pre-treatment tumor biopsy (ARIEL2 Part 1 trial) and was of high confidence based on adequate neoplastic cellularity (homozygous *BRCA1* methylation high confidence); compared with *BRCA1/2* wild-type non-*BRCA1* methylated cases, with other ever any *BRCA1*-methylated cases, and with *BRCA1/2* mutated cases.

Supplementary Table 5. List of primers for targeted amplicon sequencing of *BRCA1* and *TP53*, and *BRCA1* qRT-PCR assay.

Primer name	Primer sequence
**TP53_EX2-3_F**	tctcatgctggatccccact
**TP53_EX2-3_R**	agtcagaggaccaggtcctc
**TP53_EX4_1_F**	tgaggacctggtcctctgac
**TP53_EX4_1_R**	ctgccctggtaggttttctg
**TP53_EX4_2_F**	gtccagatgaagctcccaga
**TP53_EX4_2_R**	agaggaatcccaaagttcca
**TP53_EX5_F**	tgttcacttgtgccctgact
**TP53_EX5_R**	cagccctgtcgtctctccag
**TP53_EX6_F**	gcctctgattcctcactgat
**TP53_EX6_R**	ttaacccctcctcccagaga
**TP53_EX7_F**	cttgccacaggtctccccaa
**TP53_EX7_R**	aggggtcagaggcaagcaga
**TP53_EX8_F**	ttgggagtagatggagcct
**TP53_EX8_R**	aggcataactgcacccttgg
**TP53_EX9_F**	gacaagaagcggtggag
**TP53_EX9_R**	agtgttagactggaaacttt
**TP53_EX10_F**	caattgtaacttgaaccatc
**TP53_EX10_R**	ggatgagaatggaatcctat
**TP53_EX11_F**	agaccctctcactcatgtga
**TP53_EX11_R**	tgacgcacacctattgcaag
**PDX56_LRPCR_F**	actgaatgcaaaggacaccac
**PDX56_LRPCR_R**	ccagcaaccatttcatttcaac
**PDX19_LRPCR_F**	ccaatatcccccagtgaatg
**PDX19_LRPCR_R**	acttaggggcaaaaggcact
**HPRT1_F**	gttatggcgacccgcag
**HPRT1_R**	accctttccaaatcctcagc
**ACTB_F**	gcacagagcctcgcctt
**ACTB_R**	gttgtcgacgacgagcg
**SDHA_F**	ggacctggttgtctttggtc
**SDHA_R**	ccagcgtttggtttaattgg
**GAPDH_F**	ggtgtgaaccatgagaag
**GAPDH_R**	ccacagtttcccggag
**BRCA1_F**	gcagaggttgaagatggtatgtt
**BRCA1_R**	ccaaggcaagatctagaggga

Supplementary References

1. Gonzalez D, Collier J. DNA methylation by CcrM activates the transcription of two genes required for the division of Caulobacter crescentus. *Molecular microbiology* **88**, 203-218 (2013).

2. Topp MD*, et al.* Molecular correlates of platinum response in human high-grade serous ovarian cancer patient-derived xenografts. *Mol Oncol* **8**, 656-668 (2014).

3. Hughes-Davies L*, et al.* EMSY links the BRCA2 pathway to sporadic breast and ovarian cancer. *Cell* **115**, 523-535 (2003).

4. Jelinic P*, et al.* The EMSY threonine 207 phospho-site is required for EMSY-driven suppression of DNA damage repair. *Oncotarget* **8**, 13792 (2017).

5. Wilkerson PM*, et al.* Functional characterization of EMSY gene amplification in human cancers. *J Pathol* **225**, 29-42 (2011).
