# Supplementary material for: Methylation of all BRCA1 copies predicts response to the PARP inhibitor rucaparib in ovarian carcinoma
Source: Nat Commun. 2018 Sep 28;9:3970. doi: 10.1038/s41467-018-05564-z (PMC6162272; doi:10.1038/s41467-018-05564-z)
Supplement: Supplementary file 3 — Description of Additional Supplementary Files [file 41467_2018_5564_MOESM3_ESM.pdf]

Description of Additional Supplementary Files

File Name: Supplementary Data 1
Description: Potentially pathogenic copy number alterations, rearrangements and short variants reported by the Foundation Medicine T5a test in HGSOC PDX.

File Name: Supplementary Data 2
Description: Dose response observed in nine independent HGSOC PDX to rucaparib in vivo (150mg kg⁻¹, 300mg kg⁻¹ and 450mg kg⁻¹).

File Name: Supplementary Data 3
Description: Re-sequencing of chemo-naïve, cisplatin and rucaparib treated PDX samples from four <i>BRCA1/2</i>-mutant HGSOC for secondary mutations.

File Name: Supplementary Data 4
Description: Summary of <i>BRCA1</i> promoter methylation detected by MS-ddPCR in the archival chemo-naïve and post-treatment tumor samples in four <i>BRCA1</i>-methylated HGSOC (#11, #62, #48, #169).

File Name: Supplementary Data 5
Description: Zygosity status of <i>BRCA1</i> promoter methylation in HGSOC in relation to response to rucaparib treatment in the ARIEL2 Part 1 clinical trial.

File Name: Supplementary Data 6
Description: List of genes analysed by BROCA v6 assay.
